# Fibroblast-like synoviocytes-derived exosomal circFTO deteriorates rheumatoid arthritis by enhancing N6-methyladenosine modification of SOX9 in chondrocytes

**DOI:** 10.1186/s13075-024-03290-0

**Published:** 2024-02-22

**Authors:** Guoqing Li, Yuxuan Fang, Nan Xu, Yimin Ding, Dan Liu

**Affiliations:** 1 Department of Rheumatology and Immunology, Affiliated Hospital of Yangzhou University, Yangzhou University, Yangzhou, Jiangsu 225000 China; 2 Department of Pathology, Affiliated Subei People’s Hospital of Yangzhou University, Yangzhou, Jiangsu 225000 China

**Keywords:** Rheumatoid arthritis, Exosome, circRNA, N6-methyladenosine

## Abstract

**Background:**

Rheumatoid arthritis (RA) is a chronic inflammatory disease that causes disability worldwide. Exosomes released by fibroblast-like synoviocytes in RA (RA-FLSs-Exos) play a role in the development of RA, and circular RNAs (circRNAs) are important for RA progression. This study aimed to investigate the molecular mechanisms underlying the effects of RA-FLSs-Exos in RA and identify the potential pathway responsible for these effects.

**Methods:**

We initially conducted microarray analysis to identify dysregulated circRNAs in exosomes associated with RA. We then co-cultured isolated RA-FLSs-Exos with chondrocytes to examine their role in RA. In vivo experiments were performed using collagen-induced arthritis mouse models, and circFTO knockdown was achieved through intra-articular injection of AAV5 vectors.

**Results:**

Our findings revealed increased expression of circFTO in both RA-FLSs-Exos and synovial tissues from patients with RA. Exosomal circFTO hindered chondrocyte proliferation, migration, and anabolism while promoting apoptosis and catabolism. Mechanistically, we discovered that circFTO facilitates the formation of methyltransferases complex to suppress SRY-related high-mobility group box 9 (SOX9) expression with assistance from YTH domain family 2 (YTHDF2) through an m6A-dependent mechanism. Furthermore, inhibition of circFTO improved symptoms of RA in vivo.

**Conclusion:**

Taken together, our study demonstrates that exosomal circFTO derived from FLSs contributes to the progression of RA by targeting SOX9. These findings highlight a promising target for treating RA.

**Supplementary Information:**

The online version contains supplementary material available at 10.1186/s13075-024-03290-0.

## Introduction

Rheumatoid arthritis (RA) is a long-term autoimmune condition characterized by a systemic inflammatory response and persistent inflammation of the synovium, leading to joint swelling, synovial hyperplasia, and damage to cartilage and bone [1]. RA affects approximately 0.5-1% of adults worldwide, resulting in significant economic burdens for both society and patients [2]. Currently, the clinical treatment of RA is categorized into three groups based on the preferred medication: nonsteroidal anti-inflammatory drugs, glucocorticoids, and disease-modifying drugs [3, 4]. However, these medications exhibit resistance and lead to undesirable effects such as infections and tumors. Enhancing our comprehension of the fundamental mechanisms underlying RA pathogenesis will facilitate the development of innovative targets for RA treatment and their corresponding pharmaceutical interventions.

Research studies have demonstrated that the aberrant activation of fibroblast-like synoviocytes (FLSs) plays a pivotal role in the pathogenesis of RA [5, 6]. FLSs associated with RA secrete diverse inflammatory factors that contribute to the exacerbation of synovitis [7]. Moreover, matrix metalloproteinases (MMPs) released by RA-FLSs can induce detrimental effects on bone and cartilage within joints [8]. Furthermore, exosomes derived from RA-specific fibroblast-like synoviocytes (RA-FLSs-exo) also hold implications in the progression and pathogenesis of RA [9–11]. Exosomes are small membrane-bound vesicles ranging in size from 30 to 100 nm. They originate from various cell types and participate in immune modulation as well as immune pathogenesis associated with RA [12]. Therefore, it is imperative to investigate the potential mechanisms through which RA-FLSs-EXO regulates RA for identifying therapeutic targets for this condition.

CircRNAs, which are a type of closed circular non-coding RNAs (ncRNAs) connected by covalent bonds and possess a relatively stable structure, play a crucial role in the progression of various diseases by targeting specific genes and regulating cellular metabolism [13]. The involvement of circRNAs in RA has garnered significant attention. Analysis using microarray chips revealed the existence of a distinct expression profile for circRNAs in RA [14]. Multiple studies have confirmed the participation of numerous circRNAs in the pathogenesis of RA [15]. Yang et al. discovered that circRNA_17725 promotes M2 polarization of macrophages to alleviate arthritis symptoms [16]. Additionally, inhibiting circCDKN2B-AS_006 can mitigate the severity of arthritis by suppressing tumor-like growth and metastasis in RA-FLSs [17]. Wang et al. demonstrated that blocking the signal axis involving circPTN/miR-145-5p/FZD4 could effectively inhibit RA pathology [18]. Due to their unique expression characteristics and pivotal role in RA, circRNAs hold promise as potential diagnostic biomarkers and therapeutic targets for this condition.

Here, we explore the anomalous circRNAs in exosomes derived from RA-FLSs and found that circFTO was increased in RA-FLSs-Exo. Exosomal circFTO affected the growth, migration, apoptosis, and metabolic balance by targeting SRY-related high-mobility group box 9 (SOX9) in an N6-methyladenosine (m6A) dependent manner. Inhibition of circFTO could reduce pathological joint injury induced by CIA, indicating that circFTO may be a potentially effective therapeutic target for RA.

## Materials and methods

### Sample collection

The specimens for this study were obtained from patients at Affiliated Hospital of Yangzhou University between 2020 and 2022. Synovial tissues, collected during arthroplasty or synovectomy procedures, were sourced from a cohort of 26 RA patients (17 males and 9 females, average age: 42.34 years). Additionally, nonarthritic synovial tissues were acquired from a separate group of patients (n = 26) who underwent arthroscopy following trauma or joint derangement. Prior to study enrollment, informed consent agreement forms were duly signed. This study was carried out under the principles outlined in the Declaration of Helsinki and received approval from the Ethics Committee of the Affiliated Hospital of Yangzhou University. All participants provided written informed consent.

### Isolation of RA-FLSs

The primary RA-FLSs were obtained from synovial tissues, which were then cut into 1 mm pieces and treated with collagenase II (Gbico) at a concentration of 1 mg/ml for 4 h at a temperature of 37 °C to detach the cells. Subsequently, DMEM-F12 medium (Gbico), supplemented with 10% fetal bovine serum and 1% penicillin-streptomycin solution, was added. After centrifugation (1500 r/min, 10 min), the cells were suspended in DMEM-F12 medium and cultured at a temperature of 37 °C with 5% CO2. Cells within passages ranging from 3 to 6 were utilized for this study.

### Isolation of chondrocytes

The cartilage tissues derived from RA patients who underwent arthroplasty were sectioned into 1 mm thick slices and subsequently incubated with triploid 0.25% trypsin for a duration of 20 min. After removing the liquid portion, collagenase II was introduced at a concentration of 0.2% and incubated at 37 °C for one hour. Following centrifugation at a speed of 1000 × g for ten minutes, the cells were cultured in DMEM-F12 supplemented with fetal bovine serum (10%) and penicillin-streptomycin solution (1%). The culture medium was refreshed every three days. Chondrocytes derived from the third subculture were utilized in subsequent experiments.

### Cell transfection

The circFTO sequence was amplified and cloned into the circRNA overexpression vector GV486 (GeneChem, Shanghai, China), which consists of a front circular frame and back circular frame; an empty vector was used as a negative control. Two different shRNA were designed to suppress circFTO expression (GeneChem). SiRNA targeting Wilms tumor 1-associated protein (WTAP), methyltransferase-like 3 (METTL3), and METTL14, shRNA targeting SOX9, as well as overexpression vector for YTH domain family 2 (YTHDF2) or SOX9 were all provided by GeneChem. RA-FLSs or chondrocytes were seeded onto plates for 24 h and then transfected using Lipofectamine 3000 (Invitrogen, Carlsbad, CA, USA) following the standard protocol. The sequences of the shRNA and siRNA are provided below: sh-circFTO-1, ATGGAGGGTGTGATGATCTCA; sh-circFTO-2, GGTGTGATGATCTCAATGCCA-3′; sh-NC, 5′-TTCTCCGAACGTGTCACGTTT-3′; si-WTAP, 5′-AAGGTTCGATTGAGTGAAACA-3′; si-METTL3, 5′-CGTCAGTATCTTGGGCAAG-3′; si-METTL14, 5′-AAGGATGAACTAGAAATGCAA-3′; sh-SOX9, 5′-GGAACAACCCGTCTACACA − 3′.

### Extraction and identification of exosomes

Exosomes were isolated using a commercially available extraction kit, whereby the supernatant from P3-P6 FLSs was collected and mixed with ExoQuick TC reagent (SBI, USA), followed by overnight incubation at 4 °C. The resulting exosomes were then resuspended in PBS (100–500 µL) after centrifugation at 1500×g for 5 min to remove all liquid content. Morphological examination of exosomes was conducted using conventional transmission electron microscopy (JEM-1200EX, Hitachi, Tokyo, Japan). Particle size distribution analysis of exosomes was performed using NanoSight technology while Western blotting was employed to detect the expression of CD63 and TSG101 as markers for exosomes.

### RNA sequencing

The TRIzol reagent was used to extract RNA samples from exosomes derived from healthy FLSs (H-FLSs) or RA-FLSs, which were then subjected to sequencing by Biomarker Technologies in Beijing, China. Differential circRN screening was conducted using the R software and the negative binomial distribution test method (NB), with a threshold set at |log2-fold change (FC)| > 1 and P value < 0.05.

### Quantitative real-time PCR analysis (qRT-PCR)

The TRIzol reagent was used for extracting RNA from cells and tissues. Subsequently, the PrimeScript RT Reagent Kit was employed to perform reverse transcription for cDNA synthesis. To measure the expression of circRNA and mRNA, real-time fluorescence quantitative PCR was conducted using TB Green Fast qPCR Mix. GAPDH was utilized as an internal reference for both circRNA and mRNA. The 2^−ΔΔCt^ method was applied to analyze the expression levels of circRNA and mRNA. Please refer to supplementary Table 1 for the primer sequences utilized in PCR analysis.

### Western blotting

Western blotting analysis was performed as previously reported [19]. Antibodies used included primary antibodies against CD63 (ab134045, Abcam), TSG101 (ab125011, Abcam), WTAP (ab195380, Abcam), METTL14 (ab309096, Abcam), METTL3 (ab195352, Abcam), SOX9 (ab26414, Abcam), Ki67 (ab16667, Abcam), PCNA (#13,110, CST), BAX (#2772, CST), Cleaved Caspase-3 (#9664, CST), Cleaved Caspase-9 (#9509, CST) and GAPDH (ab8245, Abcam).

### RNA FISH

The fluorescently-labeled circFTO probe was acquired from RiboBio. FLSs were fixed in a 4% paraformaldehyde solution for 30 min and then permeabilized with 0.5% Triton X-100 for 5 min at a temperature of 4 °C. Following prehybridization, the cells were subjected to hybridization in a buffer containing specific probes overnight at 37 °C. The resulting samples were observed using confocal microscopy provided by Nikon Instruments Inc.

### Immunofluorescence (IF)

Chondrocytes inoculated on the cover glasses were fixed with a 4% paraformaldehyde solution for a duration of 15 min, followed by permeabilization using 0.25% Triton-X100 for a period of 10 min. Subsequently, the cells underwent blocking with 5% FBS for one hour and then incubation with a specific antibody at a temperature of 4 °C overnight. This was followed by incubation with an appropriate fluorescence secondary antibody. DAPI was employed as a marker for the nucleus.

### Pulldown assay with biotinylated circFTO probe

To synthesize the biotin-labeled probe for circFTO, RiboBio was utilized. A total of 2 × 10^7^ FLSs were lysed in a lysis buffer (200 µL), and after centrifugation, the supernatant was collected. From this supernatant, an aliquot of 50 µL was preserved as input while the remaining portion was mixed with either biotin-labeled antisense probes or sense probes and incubated with streptavidin C1 magnetic beads (Invitrogen) overnight at a temperature of 4 °C. The RNA-protein binding mixture underwent boiling in SDS buffer, and Western blotting was employed to detect the released proteins.

### RNA immunoprecipitation (RIP) assay

For conducting the RIP assay, we used the Magna RIP Kit from Millipore (Bedford, MA, USA). Magnetic beads were initially coated with antibodies specific to WTAP (ab195380), YTHDF2 (ab220163), m6A (ab208577), as well as corresponding immunoglobulin G (IgG), following manufacturer guidelines. These coated beads were subsequently incubated with cell lysates overnight. The extracted RNAs underwent purification for subsequent qPCR analysis, where normalization against input levels determined enrichment levels.

### RNase R resistance assay

Total RNA (2 µg) was isolated and subjected to incubation at 37 °C for one hour in both the presence and absence of RNase R (5 U/µg; Beyotime, Shanghai, China). Subsequent qRT-PCR analysis allowed us to determine levels of circFTO and FTO mRNA.

### mRNA stability assay

FLSs were cultured in 6-well plates until they reached approximately 60% confluency. Subsequently, the cells were exposed to actinomycin D at a concentration of 5 µg/mL and harvested at specified time intervals. The RNA was then isolated and subjected to qRT-PCR analysis.

### Coimmunoprecipitation (co-IP) assay

A total of 1 × 10^7^ chondrocytes were lysed using RIPA buffer containing protease inhibitors. Following a 2-hour incubation with protein A/G bead, the mixture was divided into two equal parts and subjected to overnight incubation at 4 °C with either 5 µg of WTAP (ab195380) or IgG antibody. After centrifugation and washing steps, the proteins bound to the beads were analyzed by Western blotting.

### RNA m6A quantification

Total RNA extraction from the cells was performed using TRIzol reagent (Invitrogen) according to the manufacturer’s protocol. The m6A modification levels were quantified using Abcam’s m6A RNA Methylation assay Kit (ab185912).

### Cell counting kit-8 assay

Cell viability was assessed using the Cell Counting Kit-8 assay following the manufacturer’s instructions (Dojindo). Chondrocytes were seeded in 96-well plates at a density of 5000 cells per well and incubated for 24 h, 48 h, and 72 h. Subsequently, each well received 10 µL CCK-8 solution, followed by a further incubation period of 2 h at 37 °C. The absorbance at a wavelength of 450 nm was measured using a spectrometer from Thermo Fisher Scientific.

### Transwell assay

Matrigel-coated transwell chambers with 8 μm pores were used to assess the migratory ability of chondrocytes. Specifically, a suspension of 3 × 10^4^ cells in FBS-free DMEM medium (200 µL) was added to the upper chamber, while DMEM medium containing 10% FBS was added to the lower chamber. After incubation for 24 h, cells on the top surface were removed and those that had migrated through the pores into the bottom chamber were stained with crystal violet and counted under a microscope in five randomly selected fields of view.

### TUNEL assay

To evaluate cell apoptosis, we employed an in situ Cell Death Detection Kit obtained from Boster Bio-tech (Wuhan, China), following the recommendations provided by the manufacturer.

### Adeno-associated virus preparation

The shRNA sequences for circFTO were ligated into the pro-viral plasmid (pAAV-U6-shRNA), which was then packaged into AAV5 by GeneChem (Shanghai, China). In brief, recombinant plasmids were co-transfected into HEK-293T cells along with packaged plasmids (pAAV-RC and pHelper) using the Lipofectamine 3000 (Invitrogen, Carlsbad, CA, USA). After 72 h of transfection, the transfected HEK-293T cells were subjected to centrifugation, followed by collection of the supernatant. The purified virus samples were obtained using a ViraTrap™AAV purification kit (Biomiga) and subsequently stored at -80 °C.

### Collagen-induced arthritis (CIA) mouse model

The protocols for conducting animal experiments were approved by the Institutional Animal Care and Use Committee of the Affiliated Hospital of Yangzhou University (2020-YKL03-004). All animal experiments followed the guidelines provided by the National Institutes of Health Committee on the Care and Use of Laboratory Animals from the Institute of Laboratory Animal Resources. To establish the CIA model, we utilized a previously described method [10]. Male DBA/1J mice at 8 weeks old were sourced from SLAC Laboratory Animal Co., Ltd (Changsha, China). For initial immunization, we injected a mixture of bovine type II collagen (Chondrex, Woodinville, WA, USA) and complete Freund’s adjuvant (Sigma-Aldrich, Burlington, MA, USA) into each mouse. On day 21, a booster vaccination was administered using bovine type II collagen emulsified with incomplete Freund’s adjuvant (Sigma-Aldrich, Burlington, MA, USA). We evaluated arthritis severity in each mouse using an Arthritis Index (AI), which involved scoring limb joint swelling on a scale ranging from 0 to IV: 0 indicated no redness; I represented slight swelling in toe joints; II denoted swelling in both toe and foot joints; III indicated swelling below the ankle joint; and IV signified swollen ankle joints along with other affected joints. The AI value was calculated as the total of scores assigned to all four limb joint swellings (0 level = 0 points; 1 level = 1 point; maximum score = 16 points).

For the therapeutic intervention, AAV5/sh-circFTO or AAV5/sh-NC vectors were administered on day 21, concurrently with booster vaccination. Ankle joint cavities received an injection of 5 µL of AAV5 solution containing 2 × 10^9^ viral particles per µL. This injection was repeated weekly for a duration of 5 weeks, after which the mice were euthanized 2 months following the initial administration of AAV5. To perform histology analyses, ankle and knee joints were fixed in a solution containing 4% paraformaldehyde and decalcified using a solution comprising 14% EDTA. Following dehydration, paraffin embedding was carried out to prepare paraffin sections from these joints. Routine histological staining with H&E was performed on these paraffin sections as previously described [20].

### Statistical analysis

Mean ± SD values were used to present all data. GraphPad Prism software version 8.0 was utilized for statistical analysis purposes. The data from the two groups were subjected to an independent sample t-test for comparison. For the analysis of data among multiple groups, a one-way analysis of variance (ANOVA) was conducted, followed by Tukey’s post hoc test. Statistical significance was considered when P values were less than 0.05.

## Results

### CircFTO is increased in exosomes derived from RA-FLSs

**Fig. 1:**
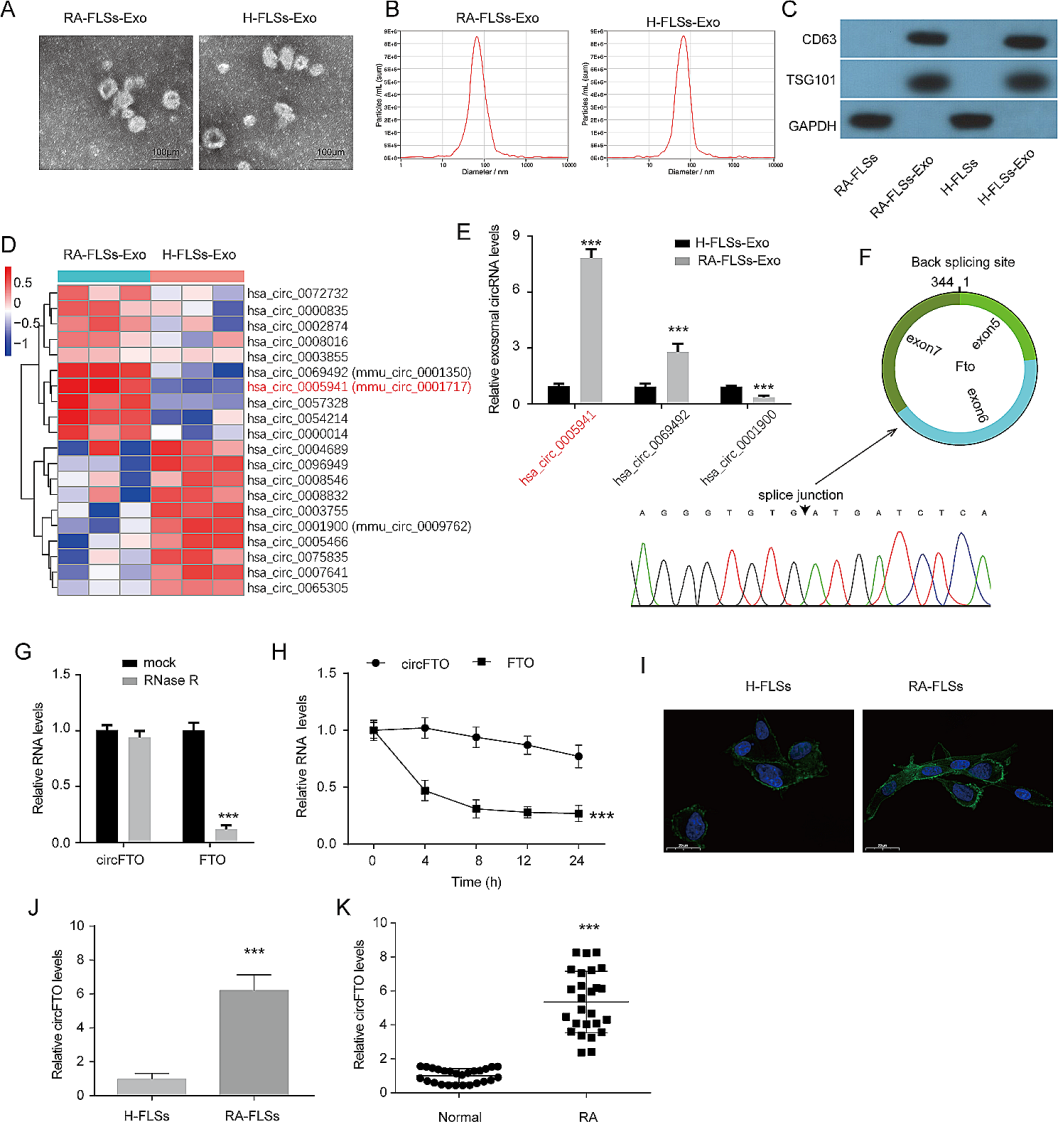
CircFTO is up-regulated in exosomes derived from RA-FLSs. (**A**) Representative images of exosomes derived from RA-FLSs and H-FLSs. (**B**) The particle size distribution of exosomes derived from RA-FLSs and H-FLSs was analyzed. (**C**) Western blot analysis was conducted to ascertain the expression levels of TSG101 and CD63, which are surface markers specific to exosomes. (**D**) A heatmap illustrating the top 10 differentially expressed circRNAs in exosomes was generated. (**E**) The expression levels of hsa_circ_0069492, hsa_circ_0005941, and hsa_circ_0001900 in exosomes were validated using qRT-PCR. (**F**) Sanger sequencing was employed to confirm the back-splicing structure of hsa_circ_0005941. (**G**) Following treatment with RNase R, qRT-PCR analysis aimed at determining circFTO and FTO expressions within RA-FSL cells was carried out. (**H**) The stability of circFTO and FTO mRNA at specified time points was assessed using an Actinomycin D assay. (**I**) The subcellular localization of circFTO in RA-FLSs and H-FLSs was determined using FISH assay. (**J**) The expression level of circFTO in both RA-FLSs and H-FSLs was quantified through qRT-PCR analysis. (**K**) qRT-PCR analysis detected the expression level of circFTO in synovial tissues from patients with RA as well as normal individuals. Data are presented as mean ± SD, n = 3, ***p < 0.001

To investigate the impact of exosomes derived from RA-FLSs on chondrocytes, we isolated exosomes from both RA-FLSs and H-FLSs. Subsequently, we characterized these isolated exosomes by examining their morphology, size, and expression of exosomal markers. Using transmission electron microscopy (TEM), we observed a series of elliptical or circular membrane vesicles ranging in size from 50 to 150 nm (Fig. 1A). The majority of secreted vesicles from both RA-FLSs and H-FLSs were approximately 100 nm in size (Fig. 1B). Furthermore, we determined the expression levels of TSG101 and CD63 as surface markers for exosomes and found that they were significantly higher in the vehicles secreted by both RA-FLSs and H-FLSs compared to their corresponding cell lysates (Fig. 1C). These results collectively confirm the successful isolation of exosomes.

The expression profile of circRNAs in exosomes derived from RA-FLSs (RA-FLSs-Exos) and H-FLSs (H-FLSs-Exos) was determined using RNA sequencing. A total of 51 up-regulated circRNAs and 46 down-regulated circRNAs were identified in exosomes derived from RA-FLSs. The top 10 up-regulated and down-regulated circRNAs were visualized in a heat map (Fig. 1D). Among these 20 aberrant circRNAs, three circRNAs (hsa_circ_0069492, hsa_circ_0005941, and hsa_circ_0001900) were highly conserved in human and mouse (sequence similarity > 85%). Subsequently, qRT-PCR was employed to validate the expression levels of these three circRNAs, revealing an increase in hsa_circ_0069492 and hsa_circ_0005941, while hsa_circ_0001900 showed a decrease in RA-FLSs-Exos (Fig. 1E). Due to its highest fold change, we selected hsa_circ_0005941 for further investigation. Hsa_circ_0005941 (chr16:53907697–53,922,863) is a circular RNA with a length of 344 bp that originates from the FTO gene. Sanger sequencing confirmed the back-splicing structure of hsa_circ_0005941 (circFTO) (Fig. 1F). Furthermore, we examined the stability of circFTO by subjecting it to RNase R digestion, which revealed its higher resistance compared to FTO mRNA (Fig. 1G). An actinomycin D assay demonstrated that circFTO has a longer half-life than FTO mRNA (Fig. 1H). FISH analysis indicated that circFTO primarily localizes to the cytoplasmic compartment both in RA-FLSs and H-FLSs (Fig. 1I). In addition, circFTO expression was also found to increase both in FSLs and synovial tissues in RA (Fig. 1J-K), Collectively, the data suggest that circFTO is overexpressed in RA-FLSs and can be secreted in an exosome-dependent manner.

### Exosomal circFTO derived from RA-FLSs promotes apoptosis, and suppresses chondrocytes proliferation and migration

**Fig. 2:**
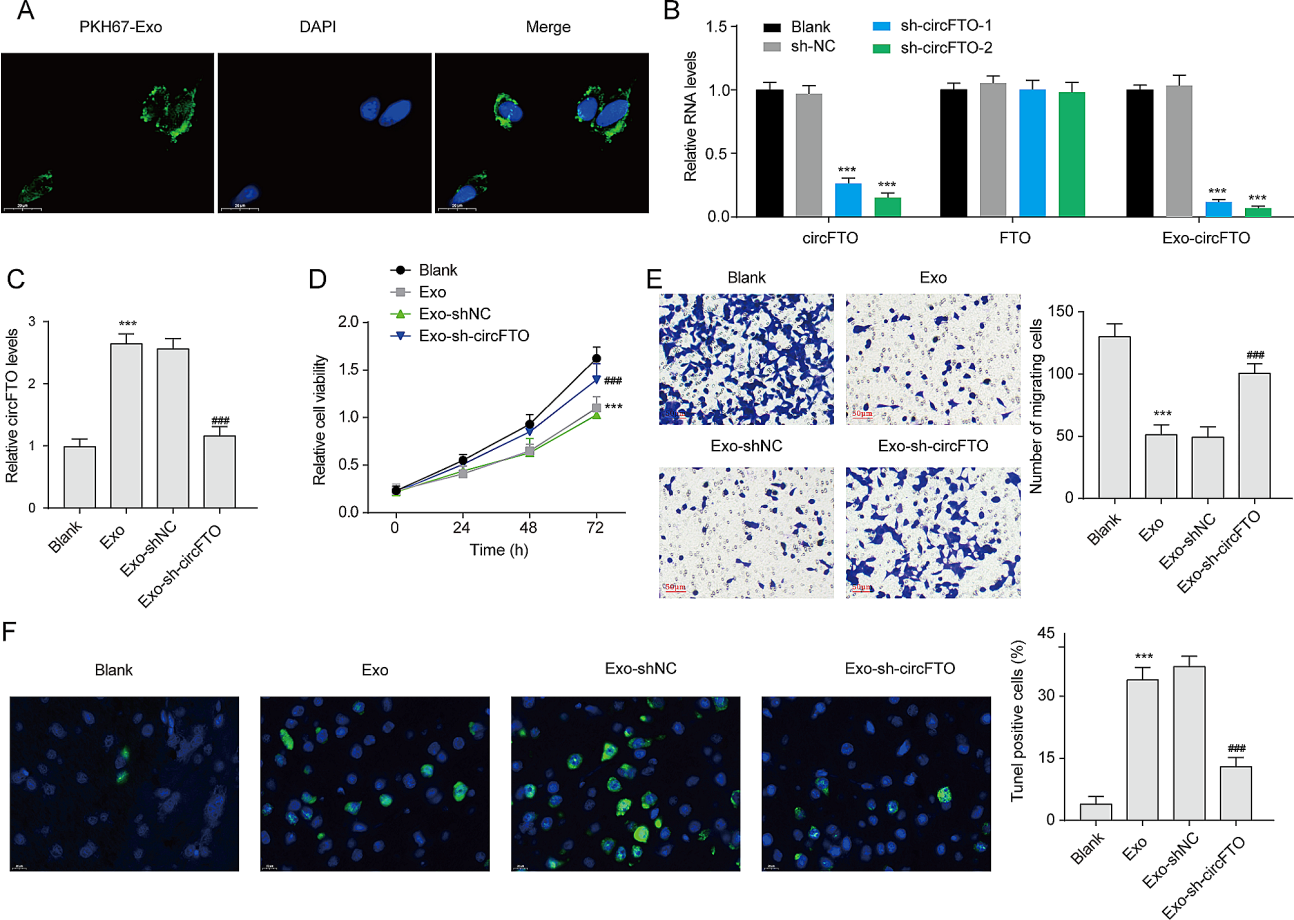
Exosomal circFTO affects the growth, migration and apoptosis of chondrocytes. (**A**) Representative images demonstrate the uptake of PKH26-stained exosomes by chondrocytes. (**B**) The expression levels of circFTO, FTO mRNA, and exosomal circFTO were assessed in RA-FLSs following the knockdown of circFTO. ***p < 0.001 vs. sh-NC. (**C**) Co-culturing with specific exosomes led to altered expression levels of circFTO in chondrocytes. (**D**) Chondrocyte viability was evaluated using a CCK-8 assay. (**E**) Transwell assay was performed to assess the migratory capacity of chondrocytes. (**F**) TUNEL assay was conducted to evaluate chondrocyte apoptosis. Data are presented as mean ± SD, n = 3; **p < 0.01, ***p < 0.001, ###p < 0.001

To investigate the impact of exosomal circFTO on chondrocytes, we co-cultured chondrocytes with exosomes derived from RA-FLSs for 48 h. For exosome tracking, we labeled the exosomes with the fluorescent dye PKH67 and observed efficient absorption of exosomes by chondrocytes (Fig. 2A). To manipulate the level of circFTO in exosomes, we specifically reduced its expression in RA-FLSs using shRNAs. Both shRNAs significantly inhibited circFTO levels in both RA-FLSs and their derived exosomes, while not affecting FTO expression in RA-FLSs (Fig. 2B). RA-FLSs-Exos notably increased circFTO levels in chondrocytes (Fig. 2C). Treatment of chondrocytes with exosomes from circFTO knockdown RA-FLSs (RA-FLS-Exos-sh-circFTO) significantly decreased circFTO expression compared to those treated with RA-FLS-Exo-sh-NC. Furthermore, reduced cell viability and migration ability were observed in chondrocytes treated with RA-FLSs-Exos (Fig. 2D-E). The knockdown of circFTO rescued the decreased cell viability and migration ability induced by these exosomes. Additionally, the TUNEL assay revealed an increase in TUNEL-positive cells among chondrocytes treated with RA-FLSs-Exos (Fig. 2F). However, the knockdown of circFTO in exosomes significantly reversed the increased number of TUNEL-positive cells, indicating that exosomal circFTO promoted the apoptosis of chondrocytes. In summary, our findings confirm that exosomal circFTO inhibits growth and migration while promoting apoptosis among chondrocytes.

### CircFTO promotes the formation of WTAP/METTL3/METTL14 complex to increase the levels of global m6A modification in chondrocytes

**Fig. 3:**
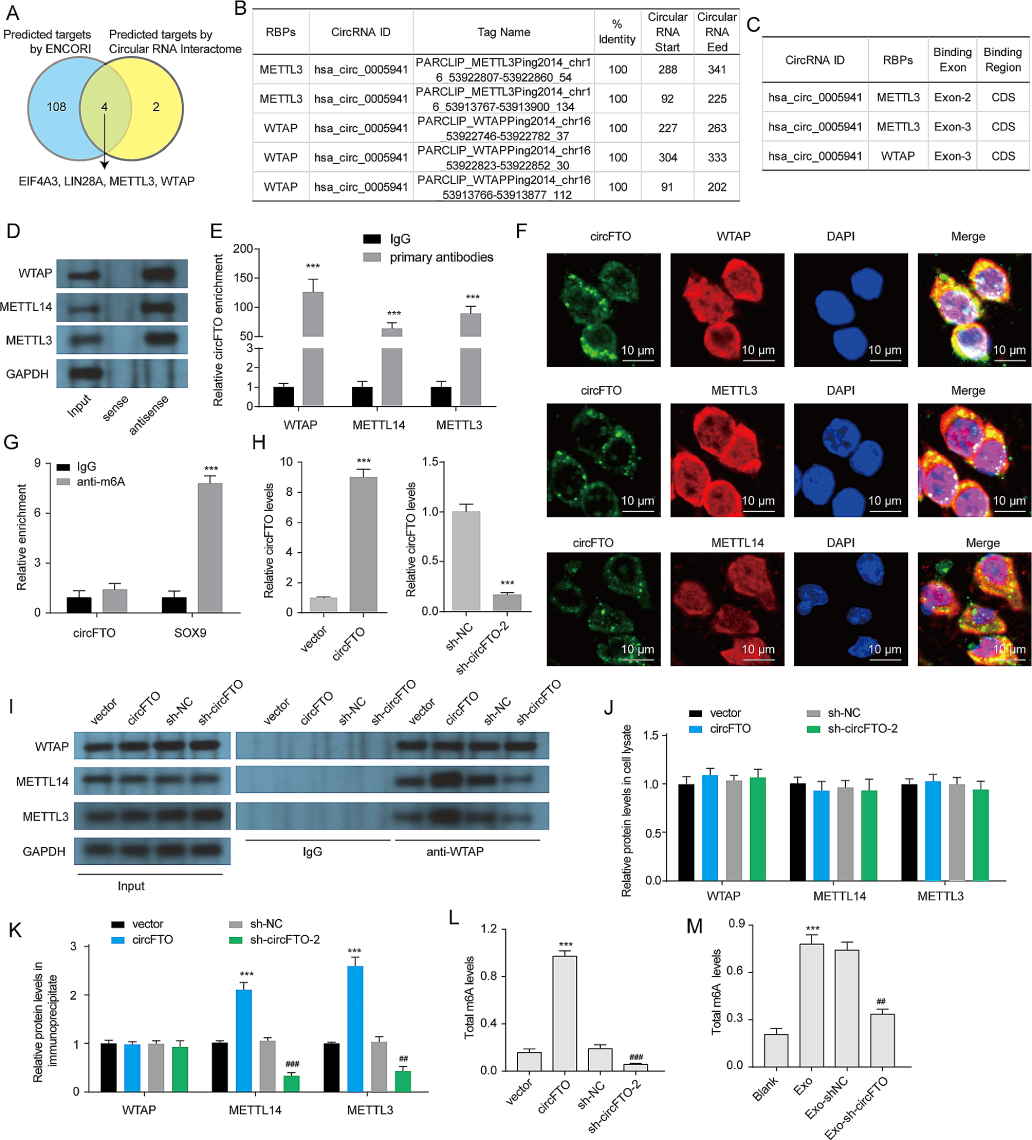
The formation of the m6A methyltransferases complex and the increase in total m6A modification levels in chondrocytes are facilitated by circFTO. (**A**) The potential RBPs were predicted by Circular RNA Interactome and ENCORI. (**B**) The Circular RNA Interactome predicted the interaction between hsa_circ_0005941 and WTAP, as well as METTL3. (**C**) ENCORI predicted the interaction between hsa_circ_0005941 and WTAP, as well as METTL3. (**D**) The interaction between circFTO and WTAP, METTL3, as well as METTL14 was assessed using a pull-down assay. (**E**) The interaction between circFTO and WTAP, METTL3, as well as METTL14 was evaluated through an RIP assay. (**F**) IF and FISH assays showing the colocalization of circFTO and WTAP, METTL3, as well as METTL14 in chondrocytes. (**G**) The level of m6A-modified circFTO was determined using an m6A RIP assay. (**H**) The expression of circFTO in chondrocytes was detected via qRT-PCR. (**I**-**K**) The impact of circFTO on the endogenous interaction between WTAP with METTL3 and METTL14 was examined using a co-IP assay. (**L**-**M**) Total m6A modification levels were quantified through an RNA m6A quantification assay. Data are presented as mean ± SD, n = 3; ***p < 0.001, ##p < 0.01, ###p < 0.001

By utilizing the Circular RNA Interactome (https://circinteractome.irp.nia.nih.gov/) and ENCORI (The Encyclopedia of RNA Interactomes, http://starbase.sysu.edu.cn/index.php), we predicted potential RBPs for circFTO. Total 4 RBPs (EIF4A3, LIN28A, METTL3, WTAP) were overlapped and predicted to bind circFTO (Fig. 3A). In these 4 RBPs, METTL3 and WTAP act as the catalytic core of methyltransferase complex (MTC) to induce m6A addition of RNA [21]. We focused on the exploration of the connection between circFTO and m6A. The potential binding sites of circFTO to WTAP and METTL3 were predicted using Circular RNA Interactome (Fig. 3B). Meanwhile, the putative binding region of WTAP and METTL3 to circFTO was determined through ENCORI analysis (Fig. 3C). Wei et al. discover a correlation between circ0008399 and WTAP, which facilitates the assembly of the WTAP/METTL3/METTL14 complex. This interaction leads to an increase in tumor necrosis factor alpha-induced protein 3 (TNFAIP3) expression through enhanced mRNA stability dependent on m6A modifications [22]. The presence of circPDE5A inhibits the WTAP-mediated m6a methylation process for eukaryotic translation initiation factor 3 (EIF3C) mRNA by forming a complex with CircPDE5-WTAP [23]. Additionally, Hu et al. propose that WTAP-mediated m6a modification contributes to improved stability for circCCAR1 [24]. Furthermore, METTL3 has been observed regulating various circRNAs such as cirRIMS2 [25], circPRKAR1B [26], and cirQSOX1 [27] through its involvement in mediating m6A methylation. Considering these findings, we hypothesized that circFTO may interact with MTC to influence self-methylation or modifying other RNAs. Subsequently, pull-down and RIP assays were conducted to validate these interactions. The biotinylated antisense of circFTO successfully pulled down WTAP and METTL3 (Fig. 3D). Furthermore, the RIP assay provided further confirmation of the interaction between circFTO and WTAP, as well as METTL3 (Fig. 3E). Moreover, FISH and immunofluorescence assay was performed to confirm that circFTO was colocalized with WTAP or METTL3 protein in chondrocytes (Fig. 3F). Especially, we also investigated the presence of METTL14 in the pull-down complex, which is not predicted to interact with circFTO but serves as a crucial catalytic component of MTC. Remarkably, the biotinylated antisense of circFTO successfully captured METTL14, while the METTL14 antibody also effectively captured circFTO. circFTO also was found to colocalize with METTL14 protein in chondrocytes. These findings strongly support the interaction between circFTO and the WTAP/METTL3/METTL14 complex.

We then investigated whether this interaction influenced the m6A modification of circFTO. Remarkably, our results from the m6A RIP assay revealed a scarcity of m6A-modified circFTO in chondrocytes; however, abundant m6A-modified SOX9 mRNA was detected in these cells (Fig. 3G). Collectively, these findings suggest that the interplay among circFTO and WTAP/METTL3/METTL14 complex does not impact the m6A modification status of circFTO. To investigate whether circFTO affects the formation of this complex, we performed co-IP assays. Our results demonstrated that overexpression or knockdown of circFTO in chondrocytes did not alter the expression levels of WTAP, METTL3, and METTL14 (Fig. 3H-J). However, overexpression of circFTO promoted the interaction between endogenous WTAP and METTL3/METTL14 proteins while knockdown of circFTO inhibited their interaction (Fig. 3K). Furthermore, we observed that overexpression of circFTO significantly increased total m6A levels in chondrocytes whereas its knockdown had an opposite effect (Fig. 3L). Additionally, exosomes derived from RA-FLSs were found to enhance total m6A levels in chondrocytes; however, knockdown of circFTO within these exosomes partially restored the total m6A levels in recipient chondrocytes (Fig. 3M), suggesting that exosomal circFTO contributes to elevated total m6A modification levels in chondrocytes. Collectively, our findings suggest a potential role for circFTO in facilitating the formation of WTAP/METTL3/METTL14 complex and enhancing overall m6A modification levels within chondrocytes.

### CircFTO reduces the expression of SOX9 in an m6A-dependent manner in chondrocytes

**Fig. 4:**
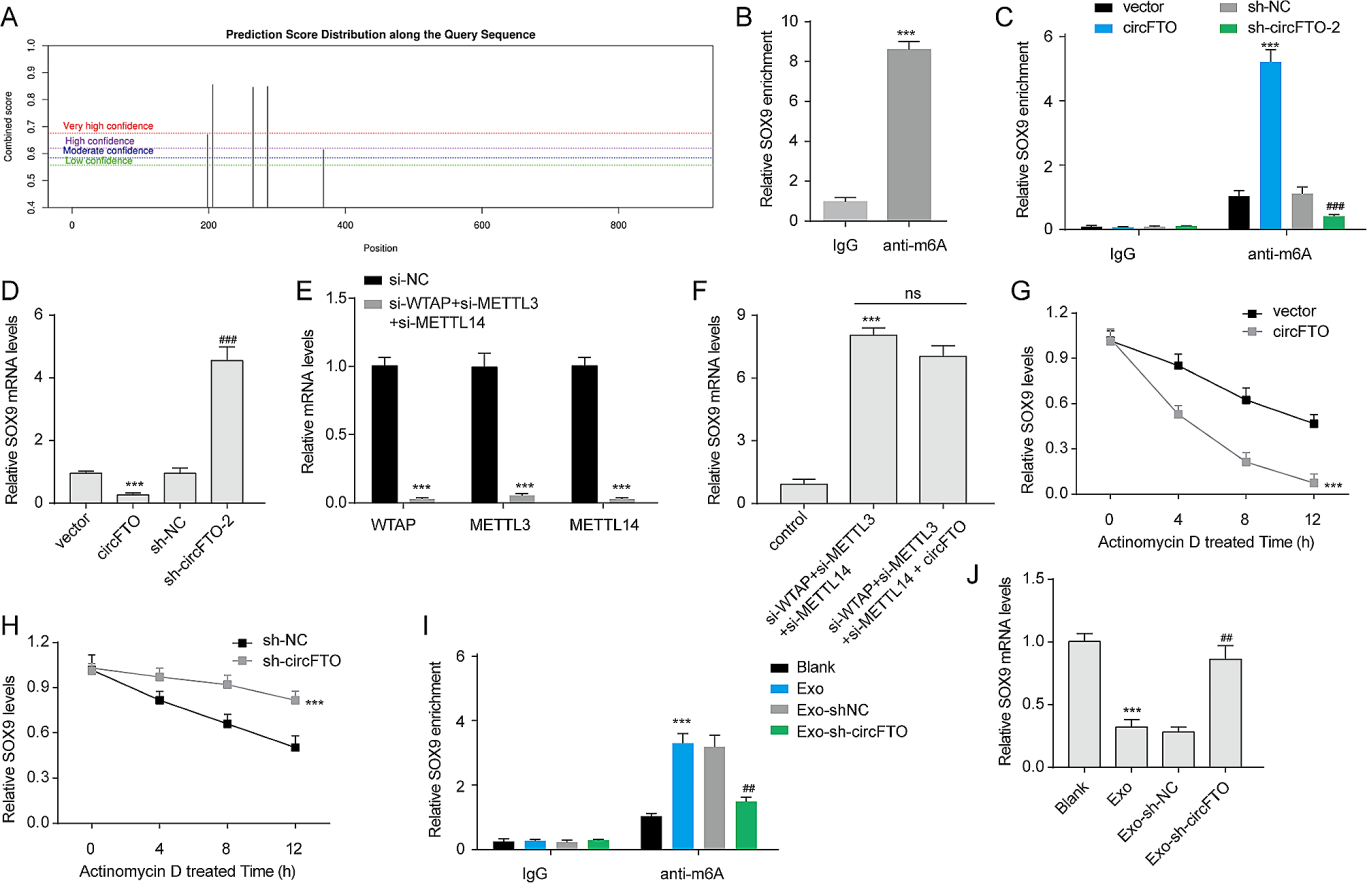
CircFTO inhibits the expression of SOX9 in an m6A-dependent manner. (**A**) Multiple potential m6A modification sites were predicted in the 3’UTR of SOX9 using SRAMP. (**B**) The level of m6A-modified SOX9 was assessed in chondrocytes through m6A RIP assay. (**C**) The impact of circFTO overexpression or knockdown on the m6A modification of SOX9 3’UTR was investigated. (**D**) The effect of circFTO overexpression or knockdown on the expression of SOX9 mRNA was examined. (**E**) The expression levels of WTAP, METTL3, and METTL14 were evaluated in chondrocytes following simultaneous knockdown of WTAP, METTL3, and METTL14. (**F**) The expression level of SOX9 was measured in chondrocytes after the indicated treatment. (**G**-**H**) The relative remaining amount of SOX9 mRNA in chondrocytes was quantified at different time points upon treatment with actinomycin D (5 µg/mL). (**I**) The influence of specific exosomes on the m6A modification status within the 3’UTR region of SOX9 was determined. (**J**) The effect of specific exosomes on the expression level of SOX9 was investigated. Data are presented as mean ± SD, n = 3; ***p < 0.001, ##p < 0.01

Previous findings have demonstrated the involvement of circFTO in the formation of the WTAP/METTL3/METTL14 complex, suggesting a potential role for circFTO in regulating RA through m6A modification of downstream target RNAs. Phenotypically, we observed that circFTO modulates chondrocyte growth, metastasis, and apoptosis. By screening the reports related to m6A and chondrocyte, we found that SOX9, a pivotal transcription factor in chondrocytes, can be affected by METTL3-mediated m6A modifications in chondrocytes [28, 29]. Considering the pivotal role of SOX9 in cartilage development and regeneration, coupled with its ambiguous involvement in RA, we identified SOX9 as a promising research target for further investigation. SRAMP (sequence-based RNA adenosine methylation site predictor, http://www.cuilab.cn/sramp) predicted multiple potential m6A modification sites in the SOX9 3’UTR (Fig. 4A). The results of m6A-RIP assay confirmed the presence of m6A modification in the SOX9 3’UTR of chondrocytes (Fig. 4B), suggesting that SOX9 may serve as a target for MTC. Previous findings indicated that circFTO could facilitate the formation of the WTAP/METTL3/METTL14 complex; therefore, we investigated whether circFTO could influence the m6A modification or expression of SOX9. Overexpression of circFTO significantly enhanced the m6A modification on SOX9 3’UTR, while depletion of circFTO markedly reduced this modification (Fig. 4C). Furthermore, overexpression of circFTO led to decreased expression levels of SOX9 mRNA, whereas knockdown of circFTO significantly increased its expression (Fig. 4D). Knockdown experiments targeting WTAP, METTL3, and METTL14 resulted in elevated levels of SOX9 mRNA and substantial reduction in WTAP, METTL3, and METTL14 expression levels (Fig. 4E-F). The overexpression of circFTO in WTAP, METTL3, and METTL14 simultaneously depleted chondrocytes had minimal impact on the expression of SOX9 mRNA, suggesting that circFTO regulates SOX9 expression by facilitating the assembly of MTC. Furthermore, circFTO overexpression significantly decreased the stability of SOX9 mRNA, while knockdown of circFTO enhanced its stability (Fig. 4G-H). We confirmed that exosomes derived from RA-FLSs elevated the level of m6A-modified SOX9 and reduced SOX9 expression in chondrocytes; however, knockdown of circFTO in exosomes partially restored these alterations (Fig. 4I-J). Therefore, these findings indicate that circFTO inhibits the expression of SOX9 through a mechanism dependent on m6A modification-mediated reduction in mRNA stability within chondrocytes.

### CircFTO-mediated inhibition of SOX9 expression is dependent on YTHDF2

**Fig. 5:**
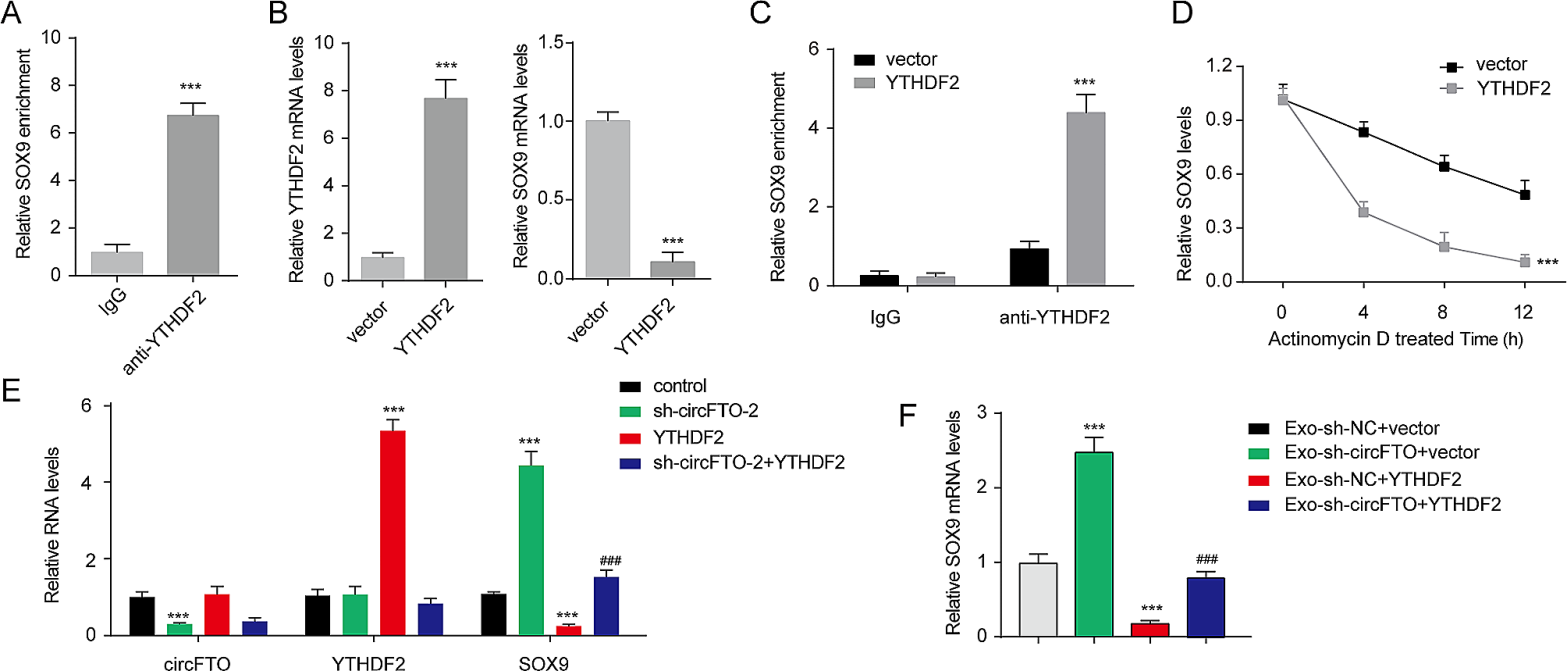
YTHDF2 is participate in regulation SOX9 expression. (**A**) The interaction between YTHDF2 and SOX9 mRNA was investigated by RIP assay. (**B**) The expression levels of YTHDF2 and SOX9 mRNA in chondrocytes were examined following YTHDF2 overexpression. (**C**) The impact of YTHDF2 overexpression on the interaction between YTHDF2 and SOX9 mRNA was assessed through the RIP assay. (**D**) The relative remaining amount of SOX9 mRNA in chondrocytes after treatment with actinomycin D (5 µg/mL) at specific time points was measured. (**E**) The expression levels of circFTO, YTHDF2, and SOX9 mRNA in chondrocytes were evaluated upon YTHDF2 overexpression or circFTO knockdown. (**F**) The expression level of SOX9 mRNA in chondrocytes was quantified. Data are presented as mean ± SD, n = 3; ***p < 0.001, ###p < 0.001

YTHDF2 has been reported to facilitate RNA degradation in an m6A-dependent manner [30]. To investigate the association between YTHDF2 and the degradation of SOX9 mRNA, a RIP assay was conducted to validate the interaction between YTHDF2 and SOX9 mRNA (Fig. 5A). Overexpression of YTHDF2 significantly upregulated its mRNA expression while downregulating SOX9 mRNA levels (Fig. 5B). Furthermore, YTHDF2 overexpression enhanced the interaction between YTHDF2 and SOX9 mRNA, leading to reduced stability of SOX9 mRNA (Fig. 5C-D). Knockdown of circFTO decreased circFTO expression, and increased SOX9 mRNA levels, but had no effect on YTHDF2 mRNA levels (Fig. 5E). Conversely, overexpression of YTHDF2 reduced SOX9 mRNA expression while increasing its transcript levels without affecting circFTO levels. Notably, overexpressing YTHDF2 rescued the promotion effect caused by circFTO knockdown on the expression of SOX9 mRNA. Additionally, exosomes depleted with circFTO-induced upregulation of SOX9 mRNA could be restored by overexpressing YTHDF2 (Fig. 5F). Therefore, it can be confirmed that circFTO/YTHDF2 regulates the expression of Sox-9.

### Exosomal circFTO regulates chondrocyte metabolic balance by targeting SOX9

**Fig. 6:**
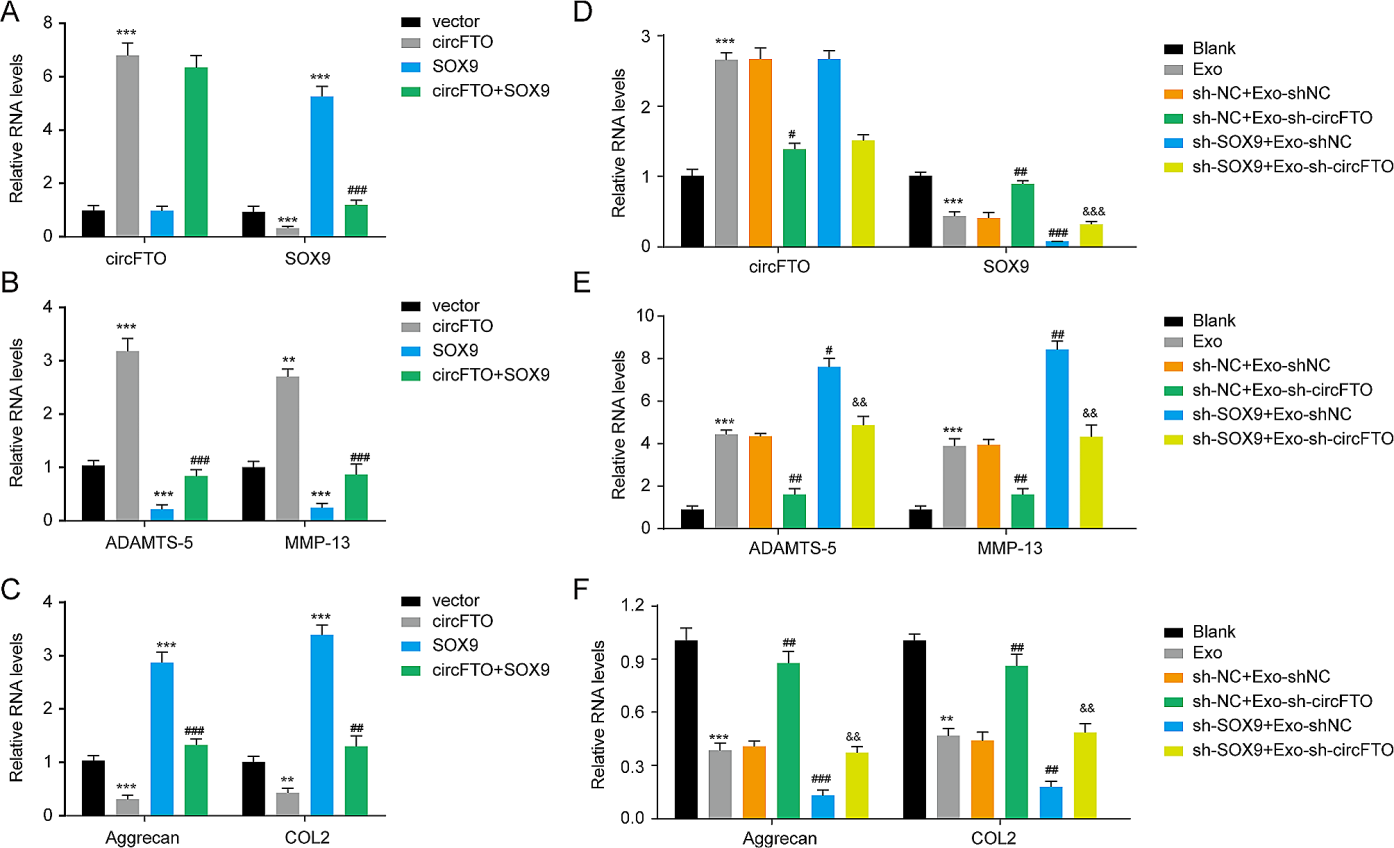
Exosomal circFTO regulates anabolism and catabolism of chondrocytes by targeting SOX9. (**A**) The expression of circFTO and SOX9 mRNA in chondrocytes following circFTO overexpression or SOX9 knockdown. (**B**) The expression of MMP-13 and ADAMTS-5 mRNA in chondrocytes following circFTO overexpression or SOX9 knockdown. (**C**) The expression of COL2 and aggrecan mRNA in chondrocytes following circFTO overexpression or SOX9 knockdown. (**D**) The expression of circFTO and SOX9 mRNA in chondrocytes after exosome treatment and transfection. (**E**) The expression of MMP-13 and ADAMTS-5 mRNA in chondrocytes after exosome treatment and transfection. (**F**) The expression of COL2 and aggrecan mRNA in chondrocytes after exosome treatment and transfection. Data are presented as mean ± SD, n = 3; **p < 0.011, ***p < 0.001, #p < 0.05, ##p < 0.01, ###p < 0.001, &&p < 0.01, &&&p < 0.001

The aberrant metabolic activities of chondrocytes are a crucial factor contributing to RA [31]. Subsequently, we investigated the impact of the circFTO-SOX9 axis on chondrocyte anabolism and catabolism. Overexpression of SOX9 reversed the inhibitory effect induced by circFTO overexpression on SOX9 levels (Fig. 6A). Notably, circFTO overexpression significantly suppressed the expression of anabolic factors (type II collagen (COL2) and Aggrecan) while increasing the expression of catabolic factors (matrix metalloproteinase-13 (MMP-13) and A Disintegrin and Metalloproteinase with Thrombospondin Motifs-5 (ADAMTS-5)) in chondrocytes (Fig. 6B-C). Conversely, SOX9 overexpression exerted opposite effects. It rescued the alterations in anabolic and catabolic factor expression induced by circFTO overexpression, suggesting that circFTO is involved in regulating chondrocyte anabolism and catabolism through targeting SOX9. Furthermore, exosomes derived from RA-FLSs enhanced catabolic factor expression while inhibiting anabolic factor expression (Fig. 6D-F). Knockdown of circFTO in exosomes restored these changes. Importantly, the knockdown of SOX9 reversed the effect of circFTO-depleted exosomes on chondrocyte metabolic balance. These findings indicate that exosomal circFTO derived from RA-FLSs can modulate chondrocyte anabolism and catabolism by targeting SOX9.

### Exosomal circFTO regulates the growth, apoptosis, and migration of chondrocytes by targeting SOX9

**Fig. 7:**
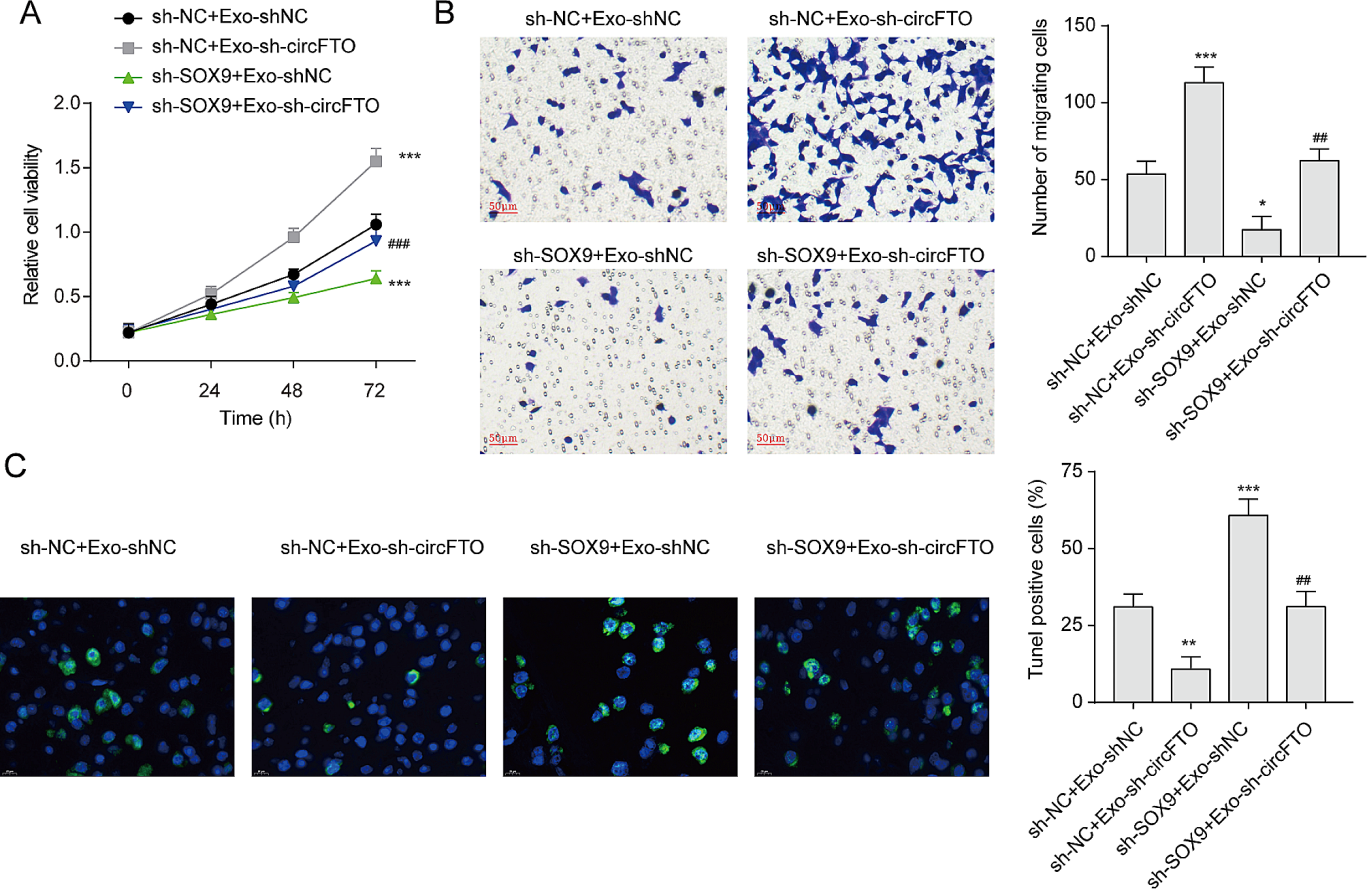
Exosomal circFTO reduces the growth and migration and promotes the apoptosis of chondrocytes by targeting SOX9. (**A**) The impact of the exosomal circFTO-SOX9 axis on chondrocyte viability was assessed using the CCK-8 assay. (**B**) The effect of the exosomal circFTO-SOX9 axis on chondrocyte migration was investigated through Transwell assays. (**C**) The influence of the exosomal circFTO-SOX9 axis on chondrocyte apoptosis was examined using TUNEL assays. Data are presented as mean ± SD, n = 3; *p < 0.05, **p < 0.01, ***p < 0.001, ##p < 0.01, ###p < 0.001

We subsequently investigated whether exosomal circFTO modulated the proliferation, apoptosis, and migration of chondrocytes by suppressing SOX9 expression. Our results demonstrated that knockdown of SOX9 significantly impaired cell viability in chondrocytes and could reverse the promotion induced by exosomes depleted of circFTO (Fig. 7A). Furthermore, a reduced number of migrated chondrocytes was observed in the group with SOX9 knockdown (Fig. 7B). The migratory effect exerted by exosomes depleted of circFTO on chondrocytes was counteracted upon knockdown of SOX9. Additionally, we found that knocking down SOX9 markedly increased the population of TUNEL-positive apoptotic chondrocytes (Fig. 7C), indicating that suppression of SOX9 promoted apoptosis in chondrocytes. Notably, inhibition of apoptosis induced by exosomes depleted of circFTO could be rescued through knockdown of SOX9, suggesting that inhibiting exosomal circFTO enhanced anti-apoptotic effects in chondrocytes via upregulation of SOX9 expression. Collectively, these findings underscore the regulatory role played by exosomal circFTO in modulating growth, apoptosis, and migration dynamics within chondrocytes through modulation of SOX9 expression.

### Knockdown of circFTO ameliorates RA in vivo

**Fig. 8:**
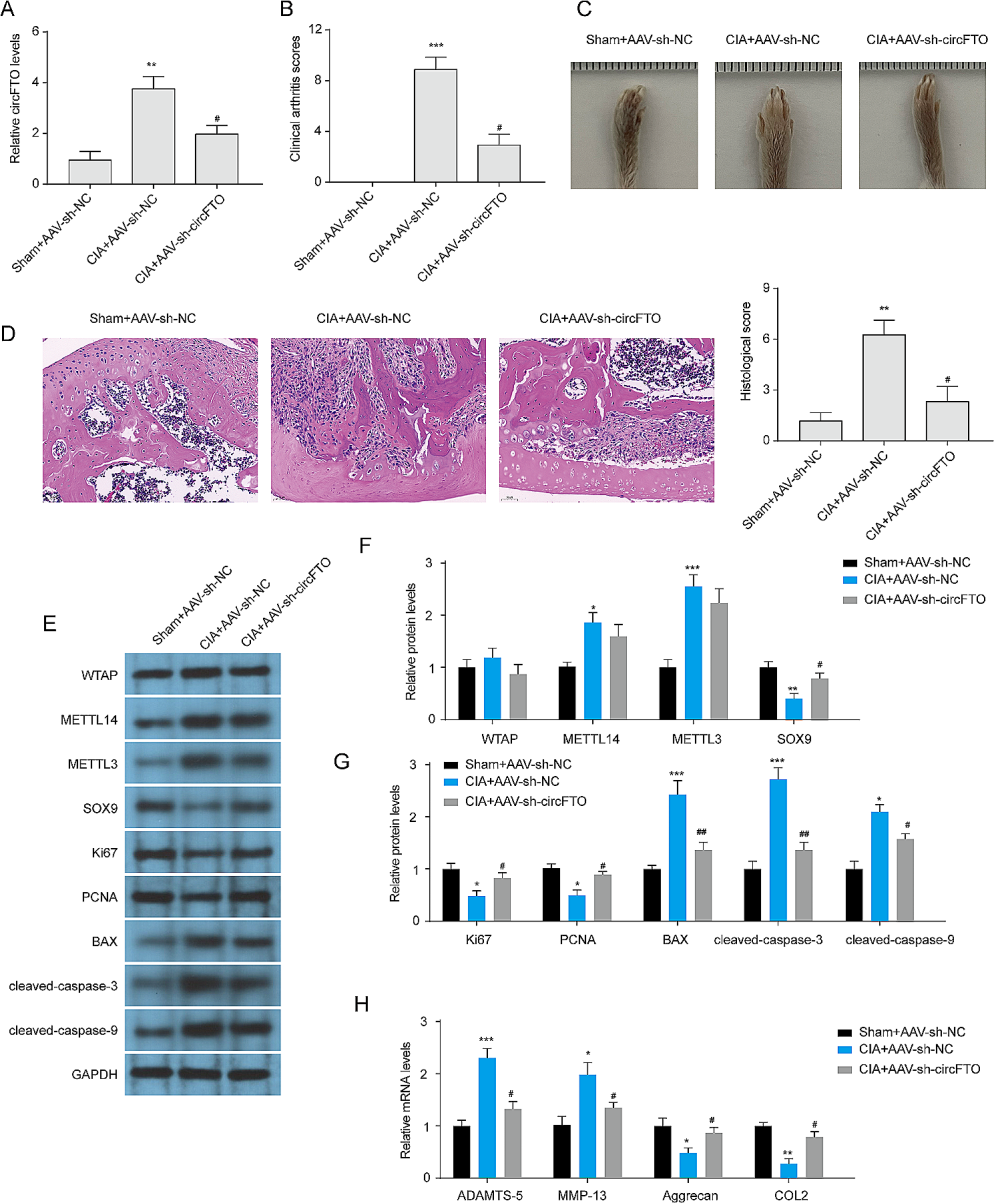
Knockdown of circFTO ameliorates RA in vivo. (**A**) The expression of circFTO in synovial tissues was quantified using qRT-PCR. (**B**) Clinical scores of CIA model mice were assessed in each experimental group. (**C**) Representative images depicting the severity of RA in CIA-induced mice were captured. 4. (**D**) Representative images and semi-quantitative analysis of hematoxylin and eosin staining were performed on cartilage tissues from CIA-induced mice. (**E**-**G**) The protein expression of WTAP, METTL14, METTL3, SOX9, proliferative markers (Ki67 and PCNA), and apoptotic markers (BAX, cleaved-caspase-3, and cleaved-caspase-9) in articular cartilage were detected by western blotting. (**H**) The mRNA expression of metabolic markers (MMP-13, ADAMTS-5, COL2 and Aggrecan) in articular cartilage was detected by qRT-PCR. Data are presented as mean ± SD, n = 6; *p < 0.05, **p < 0.01, ***p < 0.001, ***p < 0.001, #p < 0.05, ##p < 0.01

To investigate the impact of circFTO on RA progression, CIA mice were subjected to intra-articular injection of AAV5/sh-NC or AAV5/sh-circFTO. After a period of 2 months, all mice were euthanized. Notably, AAV5/sh-circFTO treatment significantly attenuated the upregulated expression of circFTO induced by CIA in synovial tissues (Fig. 8A). Moreover, administration of AAV5/sh-circFTO resulted in a distinct reduction in clinical arthritis scores among CIA-induced mice (Fig. 8B-C). Histological examination using H&E staining revealed that the sham group exhibited no evident injury and displayed normal tissue structure without any prominent inflammatory response or synovial tissue hyperplasia. Conversely, CIA mice exhibited noticeable infiltration of inflammatory cells, synovial tissue hyperplasia, and partial articular cartilage destruction (Fig. 8D). Importantly, knockdown of circFTO effectively mitigated pathological joint injury induced by CIA. Moreover, we observed a significant increase in METTL14 and METTL3 protein expression in articular cartilage upon CIA induction, accompanied by a decrease in SOX9 protein expression (Fig. 8E-F). However, WTAP expression remained unaffected. Knockdown of circFTO in CIA mice resulted in an upregulation of SOX9 protein expression in articular cartilage, while the protein expressions of WTAP, METTL3, and METTL14 were not affected. Furthermore, depletion of circFTO rescued the CIA-induced reduction in proliferative markers (Ki67 and PCNA) and elevation of apoptotic markers (Bcl-2-associated X protein (Bax), cleaved- Cysteine-aspartic protease-3 (cleaved-Caspase-3) and cleaved-Caspase-9) (Fig. 8G). Besides, circFTO knockdown restored the increased mRNA levels of MMP-13 and ADAMTS-5, as well as the decreased mRNA levels of COL2 and aggrecan in CIA mice (Fig. 8H). These data indicate that depletion of circFTO ameliorates RA in vivo by recovering SOX9 expression.

## Discussion

The limited understanding of the pathogenesis of RA hinders the development of new treatment approaches for this disease. In recent years, there has been significant interest among clinicians and researchers in exploring the potential involvement of circRNAs in RA pathogenesis. Our current investigation reveals that circFTO present in exosomes derived from RA-FLSs can impede chondrocyte proliferation, migration, and anabolism while promoting apoptosis and catabolism. Mechanistically, we demonstrate that circFTO facilitates the assembly of m6A methyltransferases complex to suppress SOX9 expression with YTHDF2’s assistance through an m6A-dependent mechanism. These findings offer valuable insights for future therapeutic strategies targeting RA.

The exosomes were obtained from FLSs and co-cultured with chondrocytes. It was discovered that exosomes derived from FLSs could hinder the growth and migration of chondrocytes while promoting their apoptosis. Extensive evidence has indicated that exosomes facilitate the direct transfer of non-coding RNAs between cells, which is considered a potential therapeutic strategy for RA [32]. Additionally, extracellular vesicles originating from FLSs have been implicated in RA. Exosomal microRNA-106b secreted by FLSs suppresses the proliferation and migration of chondrocytes in RA by reducing pyruvate dehydrogenase kinase 4 levels [11]. In vitro studies have shown that FLSs could release exosomes containing dysregulated miR-221. These exosomes interact with chondrocytes, leading to alterations in chondrocyte growth and metabolic factor levels [19]. Furthermore, exosomal miRNA-486-5p derived from FLSs in RA induces osteoblast differentiation [10]. This study proposes for the first time that exosomal circFTO originating from RA-FLSs can regulate SOX9 expression to impact various aspects such as proliferation, migration, apoptosis, anabolism, and catabolism of chondrocytes.

m6A modification, one of the important epigenetic modifications, has garnered significant attention in recent years [33]. The installation of m6A occurs through a complex of methyltransferases (“writers”) which consist of METTL3, METTL14, WTAP, KIAA1429, METTL16, RBM15 and ZC3H13 [34]. On the other hand, demethylases (“erasers”) like FTO and ALKBH5 are responsible for removing m6A modifications. Proteins that bind to m6A (“readers”), including YTHDFs and YTHDCs, possess the ability to identify RNAs modified with m6A to perform specific biological functions. Recent research has indicated that abnormalities in m6a modification are linked to various bone disorders such as osteoarthritis [35] and RA [36]. The migration, invasion, and proliferation in RA-FLSs are influenced by ALKBH5-mediated RNA methylation [37]. The involvement of METTL14-mediated m6A modification was observed in TNFAIP3 mRNA during inflammation among individuals diagnosed with active RA [38]. The presence of m6A modification on Tissue transglutaminase 2 (TGM2) mRNA has been linked to the inhibitory effects exerted by sarsasapogenin on RA-FLSs [39]. This investigation revealed that circFTO plays a facilitating role in assembling the complex responsible for m6A methyltransferases, thereby leading to an increase in overall levels of m6A within chondrocytes. Through an m6A-dependent mechanism involving YTHDF2, circFTO negatively regulates SOX9 expression by reducing its stability. These findings underscore the significance of circFTO as a key player in regulating m6A modifications associated with RA.

SOX9 is a transcription factor that plays a key role in chondrocyte reprogramming and chondrogenesis [40]. By inducing the expression of Col2 and aggrecan, SOX9 facilitates chondrocyte anabolic processes [40, 41]. SOX9 is reported to promote cartilage repair in osteoarthritis [42], whereas its role in RA still needs to be further explored. Here, we found that SOX9 is an important regulatory factor to affects the growth, migration and apoptosis of chondrocytes. SOX9 overexpression restrained catabolism and promoted anabolism of chondrocytes. The expression of SOX9 was related to the m6A modification of its mRNA in chondrocytes. Similar results also were indicated by Xiao et al. and they found that METTL3 mediated SOX9 RNA methylation and disrupted SOX9 mRNA stability [29].

## Conclusion

Collectively, RA-FLSs-derived exosomes over-expressing circFTO inhibit the proliferation and migration and promote m6a modification of chondrocytes, ultimately deteriorating RA via up-regulation of SOX9. Our finding indicates that circFTO and SOX9 can serve as potential therapeutic targets for the treatment of RA.

### Electronic supplementary material

Below is the link to the electronic supplementary material.

Supplementary Material 1

## Data Availability

The datasets used and/or analyzed during the current study are available from the corresponding author upon reasonable request.

## Abbreviations

RA: Rheumatoid arthritis
FLSs: Fibroblast-like synoviocytes
MMPs: matrix metalloproteinases
circRNAs: Circular RNAs
ncRNAs: non-coding RNAs
SOX9: SRY-related high-mobility group box 9
m6A: N6-methyladenosine
WTAP: Wilms tumor 1-associated protein
METTL3: Methyltransferase-like 3
YTHDF2: YTH domain family 2
qRT-PCR: Quantitative real-time PCR analysis
FISH: Fluorescence in situ hybridization
RIP: RNA immunoprecipitation
co-IP: Co-immunoprecipitation
CIA: Collagen-induced arthritis
MTC: Methyltransferase complex
ENCORI: The Encyclopedia of RNA Interactomes
SRAMP: Sequence-based RNA adenosine methylation site predictor
COL2: type II collagen
ADAMTS-5: A Disintegrin and Metalloproteinase with Thrombospondin Motifs-5
Bax: Bcl-2-associated X protein
Cleaved-Caspase-3: Cleaved-Cysteine-aspartic protease-3
