# Supplementary material for: Fibroblast-like synoviocytes-derived exosomal circFTO deteriorates rheumatoid arthritis by enhancing N6-methyladenosine modification of SOX9 in chondrocytes
Source: Arthritis Res Ther. 2024 Feb 22;26:56. doi: 10.1186/s13075-024-03290-0 (PMC10882813; doi:10.1186/s13075-024-03290-0)
Supplement: Supplementary file 1 — Supplementary Material 1 [file 13075_2024_3290_MOESM1_ESM.docx]

**Supplementary Table 1**

**Table S1 Data of sequences for qRT-PCR in this study.**

| **Gene** | **Sequences (5’-3’)** |
| --- | --- |
| GAPDH | Forward: GGTGGTCTCCTCTGACTTCAA |
|  | Reverse: GTTGCTGTAGCCAAATTCGTTGT |
| hsa_circ_0005941 | Forward: GTCGAGTTTGAGTGGCTGAG |
|  | Reverse: AGGTTCCTGTTGAGCACTCT |
| hsa_circ_0069492 | Forward: AGAGCCAGATGATCCACAGG |
|  | Reverse: AGGTCCTGCTATTTCTCCTCT |
| hsa_circ_0001900 | Forward: TGATGGCCTACACTGTGGAG |
|  | Reverse: CCTGCTCATACTGGTCAACG |
| FTO | Forward: ACTTGGCTCCCTTATCTGACC |
|  | Reverse: TGTGCAGTGTGAGAAAGGCTT |
| WTAP | Forward: TTCCCAAGAAGGTTCGATTG |
|  | Reverse: TGCAGACTCCTGCTGTTGTT |
| METTL3 | Forward: TGAGAACTGTTATTTCCCCATGC |
|  | Reverse: CCAGATCAGAGAGGTGGTGTAG |
| METTL14 | Forward: AGTGCCGACAGCATTGGTG |
|  | Reverse: GGAGCAGAGGTATCATAGGAAGC |
| SOX9 | Forward: CGAAATCAACGAGAAACTGGA |
|  | Reverse: ATTTAGCACACTGATCACACG |
| ADAMTS-5 | Forward: GGACCTACCACGAAAGCAGATC |
|  | Reverse: GCCGGGACACACGGAGTAC |
| MMP-13 | Forward: TGGAAGGATGCCTTTTTTTCTC |
|  | Reverse: CACCCTCCCCAAGTATCAATAGG |
| COL2 | Forward: AGGGCAACAGCAGGTTCACATAC |
|  | Reverse: TGTCCACACCAAATTCCTGTTCA |
| Aggrecan | Forward: AAATATCACTGAGGGTGAAGCCCG |
|  | Reverse: ACTTCAGGGACAAACGTGAAAGGC |
| YTHDF2 | Forward: AGCCCCACTTCCTACCAGATG |
|  | Reverse: TGAGAACTGTTATTTCCCCATGC |
